# Mucosal cell populations may contribute to peripheral immune abnormalities in HIV-infected subjects introducing cART with moderate immune-suppression

**DOI:** 10.1371/journal.pone.0212075

**Published:** 2019-02-14

**Authors:** Matteo Basilissi, Camilla Tincati, Esther Merlini, Giuseppe Ancona, Elisa Borghi, Francesca Borgo, Alessandra Barassi, Antonella d’Arminio Monforte, Giulia Marchetti

**Affiliations:** 1 Department of Health Sciences, Clinic of Infectious Diseases, ASST Santi Paolo e Carlo, University of Milan, Italy; 2 Department of Health Sciences, Microbiology Laboratory, University of Milan, Italy; 3 Department of Health Sciences, Biochemistry Laboratory, ASST Santi Paolo e Carlo, University of Milan, Italy; University of Pittsburgh Centre for Vaccine Research, UNITED STATES

## Abstract

HIV infection causes the progressive depletion of CD4+ T-lymphocytes and profound modifications of T-cell homeostasis, which persist despite virologically-suppressive treatment and have been linked to a worse clinical outcome. Enduring alterations of the gastrointestinal tract may represent the underlying pathogenic mechanisms of these phenomena. Twenty-six HIV-infected subjects were assessed over a 12-month period following the introduction of antiretroviral therapy. 18 uninfected individuals were enrolled as controls. Parameters of peripheral T-cell homeostasis (activation, maturation), gastrointestinal function (microbial translocation, gut inflammation, fecal microbiota composition) and mucosal immunity (CD4+CCR6+CD161+, CD4+CCR9+α4β7+, stem cell memory CD4+/CD8+ T-cells) were assessed. CD4+CCR6+CD161+ cells were depleted in HIV-infected untreated subjects and maintained significantly lower levels compared to controls, despite the introduction of effective antiviral treatment. The frequency of gut-homing CD4+CCR9+α4β7+ cells was also impaired in untreated infection and correlated with the HIV RNA load and CD4+HLADR+CD38+; during therapy, we observed a contraction of this pool in the peripheral blood and the loss of its correlation with antigenic exposure/immune activation. A partial correction of the balance between stem cell memory pools and T-cell homeostasis was registered following treatment. In HIV-infected subjects with moderate immune-suppression, antiretroviral therapy has a marginal impact on mucosal immune populations which feature distinctive kinetics in the periphery, possibly reflecting their diverse recruitment from the blood to the mucosa. The persistent defects in mucosal immunity may fuel peripheral T-cell abnormalities through diverse mechanisms, including the production of IL-17/IL-22, cellular permissiveness to infection and regulation of T-lymphocyte maturation.

## Introduction

Combination antiretroviral therapy (cART) suppresses HIV viral load leading to increases in CD4+ T-cell counts, yet T-lymphocyte homeostasis invariably remains impaired, with the expansion of activated/exhausted T-cell subsets and contraction of the naïve/memory ratio[1–6]. Importantly, the persistence of such defects has been linked to the lack of immunologic recovery as well as the development of non-AIDS comorbidities in the setting of viral suppression[7–12].

Considerable evidence exists on the impairment of the gastrointestinal tract during HIV infection, determining disease pathogenesis and clinical outcome[13–17]. Ensuing studies allowed for the identification and investigation of cell populations involved in gut health, shedding light on the kinetics and mechanisms of their loss in the course of HIV infection, and cART-mediated reconstitution[18–23]. In contrast, a limited number of researches, conducted mainly in cross-sectional studies enrolling heterogeneous populations in terms of CD4+ count and cART length, addressed whether a link between mucosal cell populations, persistent defects in peripheral T-cell homeostasis and disease outcome exists in the context of treated HIV disease[24–32].

Our study followed antiretroviral-naïve subjects with moderate immune-suppression for 12 months after cART introduction to explore the association between T-cell maturation/activation, parameters of gastrointestinal function (microbial translocation, gut inflammation, fecal microbiota composition) and mucosal immunity (CD4+CCR6+CD161+, CD4+CCR9+α4β7+, stem cell memory CD4+/CD8+ T-cells, Tscm).

## Material and methods

The Ethics Committee of our Institution approved the study and the written informed consent which was obtained from all participants. No minors were included in the study.

### Study participants

HIV-infected, antiretroviral-naïve subjects introducing cART (T0) were consecutively recruited at the Clinic of Infectious Diseases and Tropical Medicine, ASST Santi Paolo e Carlo, University of Milan, Italy. Participants were followed-up and included in the present study if HIV RNA load was undetectable (HIV RNA <40 copies/ml) after 12 months of treatment (T12). HIV-uninfected age- and sex-matched individuals were selected as controls.

### Human lymphocyte separation and flow cytometry surface staining

Cryopreserved PBMCs collected at T0 and T12 were thawed and stained (1x10^6^ cells) with fluorochrome-labelled antibodies for the flow cytometric study of lymphocyte surface phenotypes. To check cell viability, cells were stained with 7-aminoactynomycin D (7-AAD, BD Biosciences, San Jose, California, USA) for 30 min in the dark at 4°C. Only samples with cellular viability greater than 70% were used for experiments.

The following antibodies were used: HLA-DR-FITC, CD38-PE, CCR7-PeCy7, CD45RA-PeCy5, CD27-PE, CD95-APC, α4β7integrin-APC CCR6-PeCy7, CD161-APC (BD Biosciences, San Jose, California, USA), CCR9-FITC (R&D Systems, Minneapolis, MN, USA).

We evaluated CD4+ and CD8+ activation (HLA-DR+CD38+), maturation (naïve: CCR7+CD45RA+; central memory: CCR7+CD45RA-; effector memory: CCR7-CD45RA-; terminally differentiated: CCR7-CD45RA+) and stem cell-like memory T cells (Tscm; CCR7+CD45RA+CD27+CD95+). CD4+ T-cell populations involved in mucosal immunity (CCR9+α4β7+; CCR6+CD161+) were also studied.

Cells were run on a FACS VERSE cytometer (BD Biosciences, San Jose, California, USA).

### Microbial translocation parameters and fecal calprotectin quantification

Plasma soluble CD14 (sCD14) and Endotoxin core Antibodies (EndocAb) were measured by ELISA (R&D Systems, Minneapolis, Minnesota, USA), in accordance with the manufacturer’s instructions. Samples were diluted 1000 times. Circulating lipopolysaccharide (LPS) was assessed using the Lymulus Amebocyte Lysate (LAL) test (Lonza Group Ltd, Basel, Switzerland), as per manufacturer’s instructions. Samples were diluted 1:150 and preheated at 95°C for 10 min.

Fecal calprotectin was tested by ELISA (PhiCal, Eurospital, Italy).

### Fecal microbial population analyses

Feces were collected at T0 and T12, frozen at -20°C until use. Total bacterial DNA was extracted from 200 mg of feces using the PSP Spin Stool DNA Plus kit (Stratec Molecular, Berlin, Germany).

Analysis of the microbial population was executed as previously described [17] by denaturing gradient gel electrophoresis (DGGE) (PhorU system, Ingeny, Netherlands. The bacterial taxa reported in literature with a key-role in inflammation and gut permeability-modification were quantified through Real Time PCR using StepOne method (Applied Biosystems, USA); hence we selected four genera (*Lactobacillus*, *Roseburia*, *Bacteroides* and *Prevotella*) and one family (*Enterobacteriaceae*) for statistical analyses.

### Statistical analysis

Data were analyzed with GraphPad 6 PRISM software (GraphPad Inc., La Jolla, California, USA). Fisher’s exact test, Chi-squared test, Mann-Whitney *U*-test, Wilcoxon signed rank test and Spearman correlation were used for statistics. Differences were considered statistically significant at p< 0.05.

## Results

### Patient population

Twenty-six antiretroviral-naïve HIV-infected subjects were consecutively enrolled (T0) and followed for 12 months after cART introduction (T12). At baseline, median HIV RNA load, CD4+ T-cell counts, and CD4+/CD8+ ratio were log_10_ 4.7 (IQR 4.2–5.3), 366 cells/ul (IQR: 273–428) and 0.3 (IQR 0.2–0.4), respectively (Table 1). Following treatment, all subjects presented viral suppression (log_10_ HIV RNA: 1.6, IQR 1.6–1.6; p = 0.0001), a significant recovery in CD4+ T-cell numbers (477 cells/mmc; IQR 269–589; p = 0.0001) and increase of the CD4+/CD8+ T-cell ratio (0.5, IQR 0.4–0.6; p = 0.0001). Eighteen HIV-uninfected age- (age: 33 years, IQR 29–38; p = 0.08) and sex-matched individuals (females: n = 3, 19%; p = 0.8) were enrolled as controls.

**Table 1:** Demographic and clinical characteristics of the study population.

**Parameter at baseline**	**HIV-infected subjects (n = 26), T0**	
**Sex, F (%)**	4 (15%)	
**Age, years (IQR)**	39 (32–45)	
**Risk factors for HIV infection, n (%)**		
Heterosexual	8 (31)	
MSM	17 (65)	
IDU	1 (4)	
**HCV co-infection n (%)**	3 (12)	
**HBV co-infection**	1 (4)	
**Co-trimoxazole use, n (%)**	3 (12)	
**Duration of HIV infection, months (IQR)**	16 (4–52)	
**AIDS diagnosis, n (%)**	2 (7)	
**First cART regimen n, (%)**		
PI+NRTI	7 (27)	
NNRTI+NRTI	14 (54)	
Other	5 (19)	
**Parameter in the course of the study**	**HIV-infected subjects (n = 26)**	
	**T0**	**T12**
**HIV RNA Log**_**10**_ **cp/mL (IQR)**	4.7 (4.2–5.3)	1.6 (1.6–1.6)*
**CD4+ T-cell count, cell/mmc (IQR)**	366 (273–428)	477 (269–589)*
**CD4+ T-cell, %(IQR)**	18 (15–22)	25 (21–31)
**CD8+ T-cell count, cell/mmc (IQR)**	1018 (853–1384)	949 (806–1304)
**CD8+ T-cell, %(IQR)**	56 (53–60)	51 (44–58)
**CD4+/CD8+ T-cell ratio (IQR)**	0.3 (0.2–0.4)	0.5 (0.4–0.6)*

Data are presented as median, interquartile range (IQR) for continuous variables; absolute number, percentage for categorical variables. MSM: Men having Sex with Men; IDU: Intravenous Drug Use; HCV, Hepatitis C Virus, infection defined as the presence of detectable plasma HCV RNA; HBV, Hepatitis B Virus, infection defined as HBsAg positivity; AIDS: Acquired Immune Deficiency Syndrome; cART Combination of Antiretroviral Therapy; NNRTI, non-nucleoside transcriptase inhibitor; NRTI, nucleoside transcriptase inhibitor, PI, protease inhibitor. Data were analyzed by Fisher’s exact, Wilcoxon and Mann—Whitney tests where appropriate.

*indicates p<0.0001 for T0 vs T12.

### Persistence of microbial translocation and gut inflammation and only partial modification of the fecal microbiota and CCR6+CD161+ cell frequencies in HIV-infected subjects introducing cART

**Fig 1:**
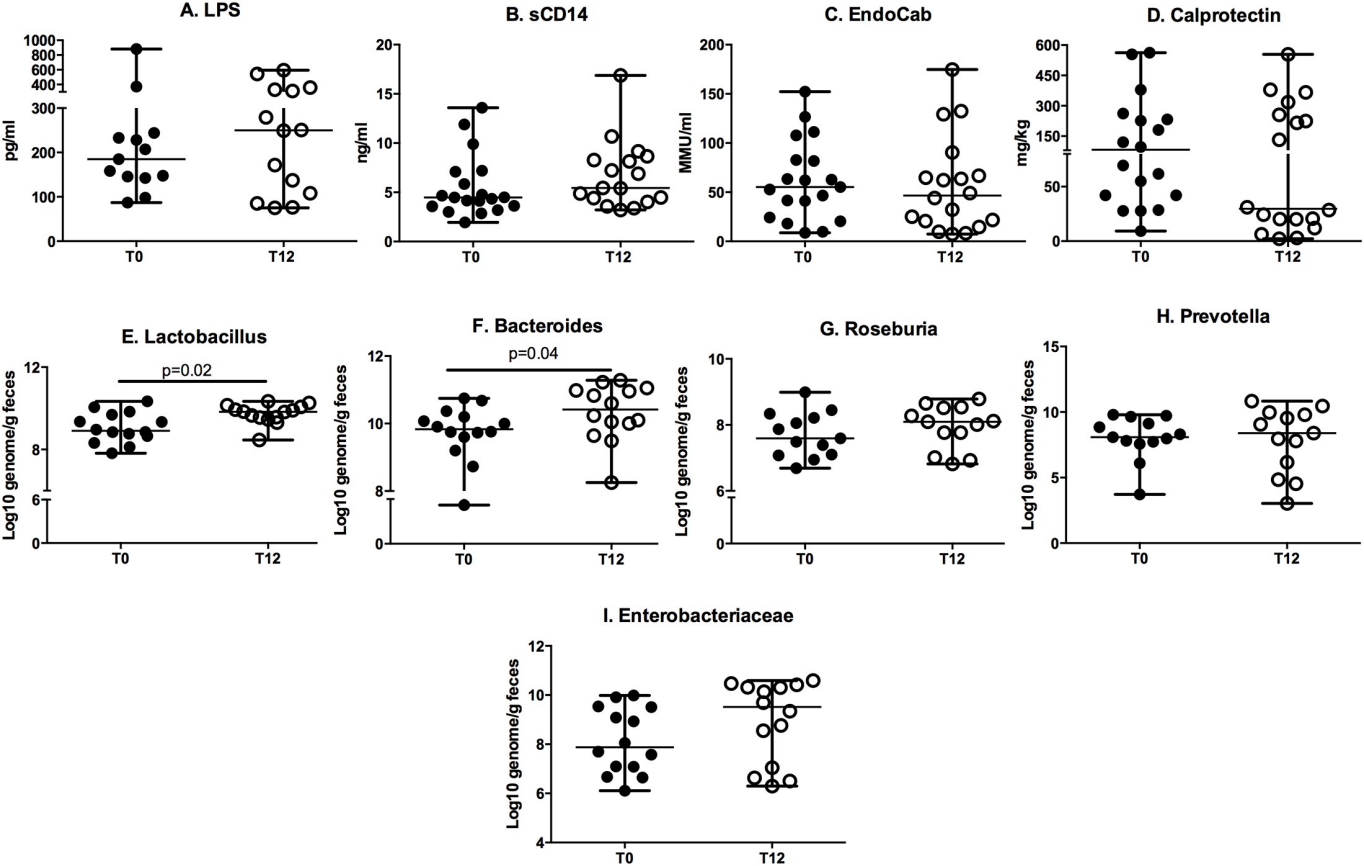
Microbial translocation, gut inflammation and faecal microbiota composition in HIV-infected subjects prior to (T0) and following 12 months of cART (T12). No significant variation in microbial translocation and gut inflammation parameters was measured in the course of the study, while a selective increase of Lactobacillus and Bacteroides was detected among fecal bacteria genera. LPS, lipopolysaccharide; sCD14, soluble CD14; EndocAb, Endocore toxin Antibodies. Data are presented as median, interquartile range (IQR) and were analyzed by Wilcoxon signed rank test.

No differences were detected in terms of microbial translocation (LPS, sCD14, EndocAb; Fig 1A–1C) or gut inflammation parameters (calprotectin; Fig 1D) prior to and following treatment.

No changes were registered in terms of fecal microbiota composition upon DGGE analysis (not shown). When quantifying bacterial taxa, we found a significant increase of the *Lactobacillus* (phylum *Firmicutes*) and *Bacteroides* (phylum *Bacteroidetes*) genera (Fig 1E and 1F), whereas no modifications of *Roseburia* (phylum *Firmicutes*) and *Prevotella* (phylum *Bacteroidetes*) genera (Fig 1G and 1H) nor of the *Enterobacteriaca*e family (phylum *Proteobacteria*) (Fig 1I).

**Fig 2:**
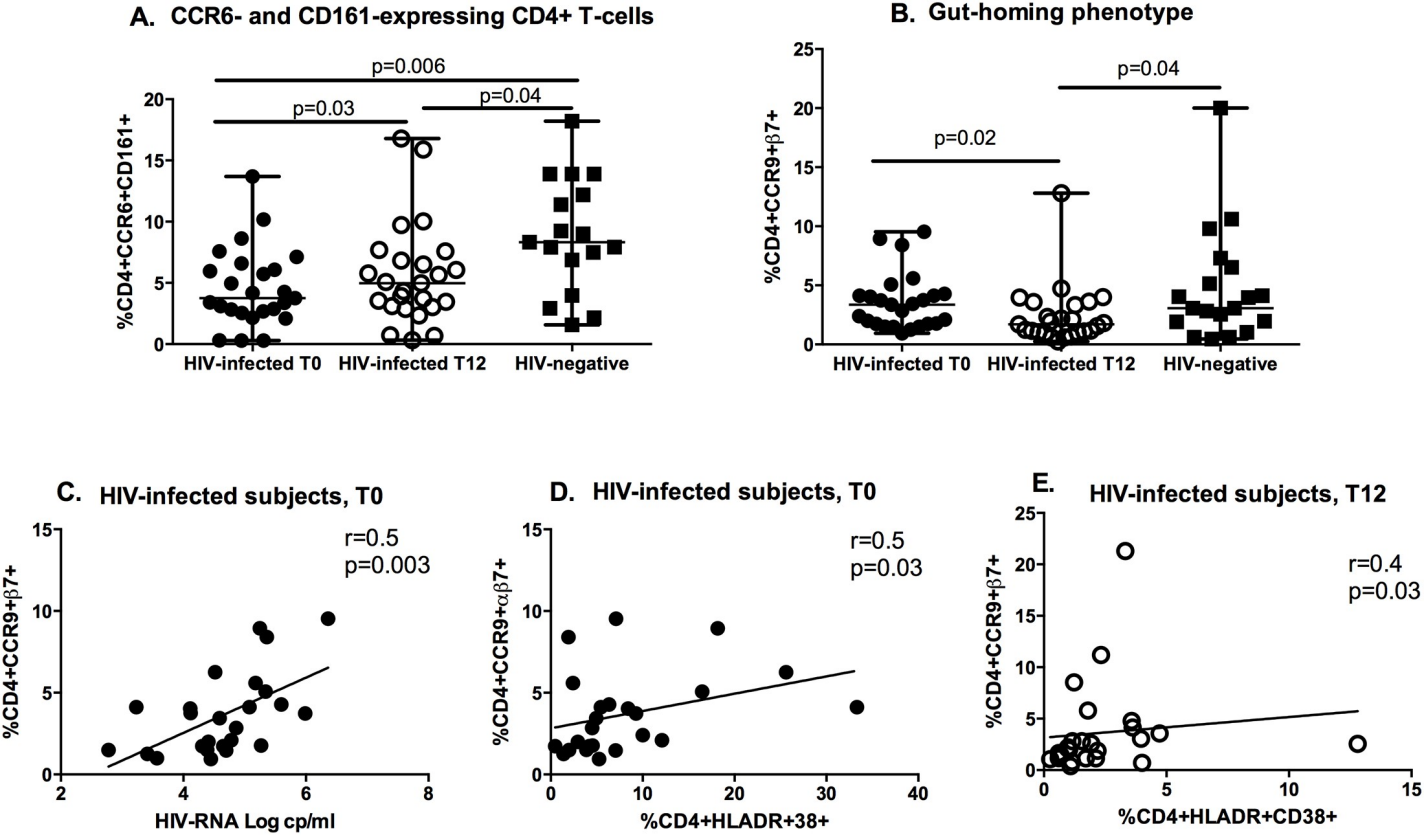
Frequency of CD4+CCR6+CD161+ (A) and CD4+CCR9+α4β7+ (“gut homing”) cells (B) and their correlation with viral load (C) and immune activation parameters (D, E) prior to (T0) and following cART (T12). CD4+ T-cell subsets expressing CCR6+CD161+ significantly increased in the peripheral blood following antiretroviral therapy, while those expressing CCR9+α4β7+ decreased upon cART. Pre-cART viral load and CD4+ T-cell activation both positively correlated with gut-homing CD4+ T-cells. Data are presented as median, interquartile range (IQR). Changes in study parameters in HIV-infected subjects introducing cART were measured by Wilcoxon signed rank test; comparisons between HIV-infected and uninfected individuals were assessed by Mann-Whitney test. Correlations were analyzed by Spearman’s Correlation test.

In line with the finding of persistent dysbiosis in treated infection, the CD4+CCR6+CD161+ subset, which may protect the intestinal mucosa through the production of IL-17 and IL-22 [33–35], increased in the course of cART (T0: 3.8% IQR: 2.6–6.3; T12: 5% IQR: 3.1–7.2; p = 0.03; Fig 2A), yet maintained a significantly lower frequency compared to HIV-uninfected controls (CCR6+CD161+ in HIV-: 8.3% IQR: 5.4–13.1; see above for HIV+; p = 0.04; Fig 2A).

### Persistent impairment of “gut-homing” and Tscm in HIV-infected subjects introducing cART

In HIV-infected, untreated patients, we report lower frequencies of CD4+ T-cells with a “gut-homing” (CCR9+α4β7+) phenotype compared to HIV-uninfected controls (HIV+: 3.4% IQR: 1.7–4.2; HIV-: 3.1% IQR: 1.7–6.7; p = 0.7 Fig 2B). A further decrease was shown after 12 months of cART (T12: 1.7% IQR: 1–3.5; p = 0.02; Fig 2B). A positive correlation was found between this subset and the levels of plasma HIV RNA (r = 0.5; p = 0.003; Fig 2C) and activated CD4+HLA-DR+CD38+ (r = 0.5, p = 0.03; Fig 2D) prior to cART introduction.

**Fig 3:**
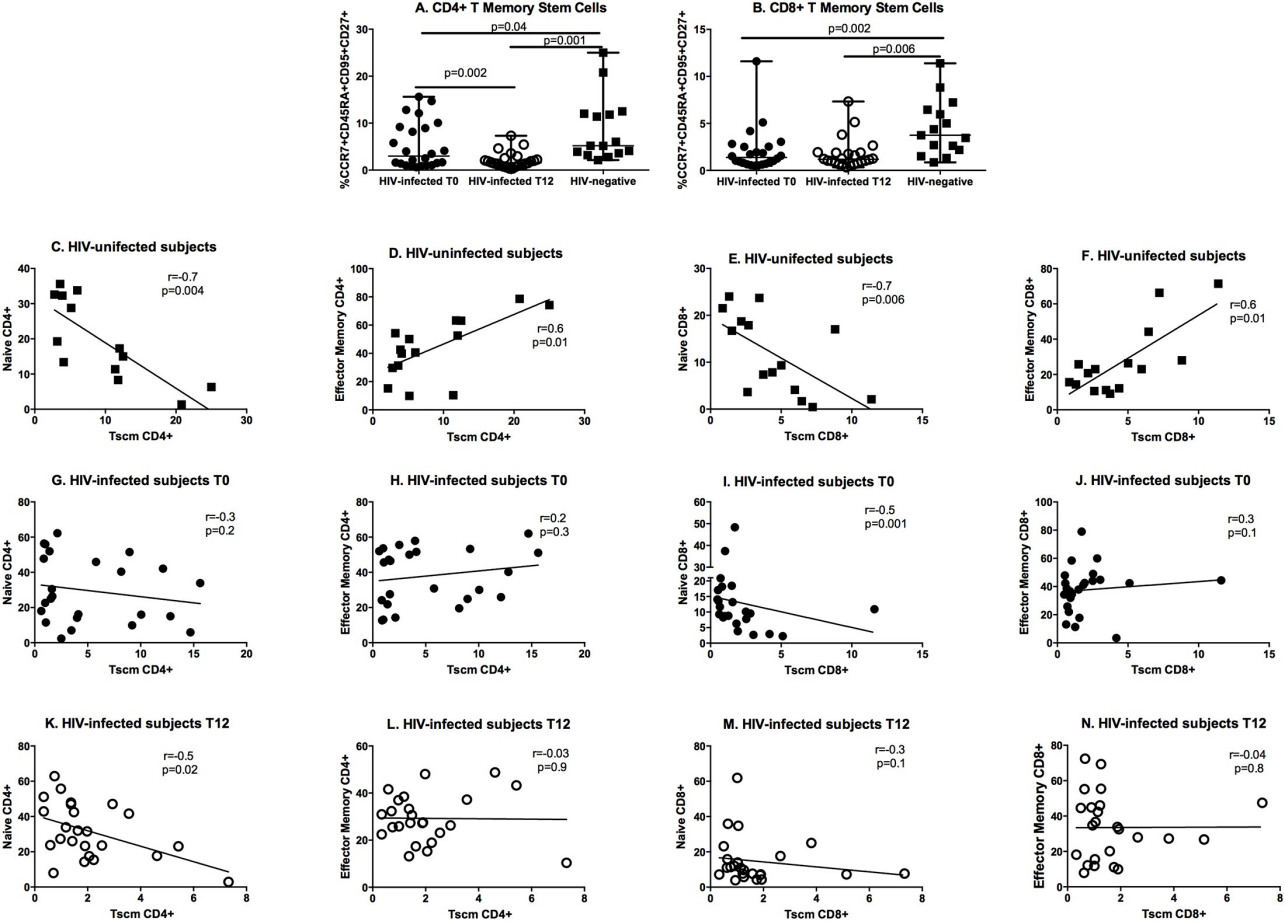
CD4+ (A) and CD8+ (B) Tscm frequencies and their correlation with maturation subsets in HIV-uninfected (C-F) and HIV-infected infected subjects prior to (T0; G-J) and following cART (T12; K-N). A significant reduction of the CD4+ Tscm pool and no changes in the CD8+ Tscm subset was measured in the course of the study and both maintained lower frequencies compared to controls. In HIV-negative individuals, Tscm cells correlated negatively with naïve and positively with effector memory subsets. This relationship lacked in HIV-infected, untreated individuals and was partially restored in the course of cART. Data are presented as median, interquartile range (IQR) for continuous variables. Changes in study parameters over time in HIV-infected subjects introducing cART were measured by Wilcoxon signed rank test; comparisons between HIV-infected and uninfected individuals were assessed by Mann-Whitney test. Tscm, T stem cell memory cells. Correlations were analyzed by Spearman’s Correlation test.

HIV infection accounted for lower CD4+ and CD8+ Tscm frequencies compared to uninfected controls (CD4+ Tscm; HIV+, T0: 2.9% IQR: 1.1–9.1; HIV-: 5.2% IQR: 3.6–12; p = 0.04; Fig 3A; CD8+ Tscm; HIV+, T0: 1.4% IQR: 0.7–2.5; HIV-: 3.7% IQR: 2.2–6.5; p = 0.002; Fig 3B). We describe a significant reduction of the CD4+ Tscm subset in HIV-infected subjects during the first 12 months of cART (T12: 1.6% IQR: 1.0–2.5; p = 0.002; Fig 3A) and no variations of the CD8+ Tscm pool (T12: 1.2% IQR: 0.8–1.9; p = 1; Fig 3B). The net result of these changes was the persistent impairment of the CD4+ and CD8+ Tscm pools in HIV-infected subjects compared to controls (p = 0.001 and p = 0.006 respectively; Fig 3A and 3B).

In uninfected controls, Tscm correlated negatively with naïve (CD4+: r = -0.7; p = 0.004; Fig 3C; CD8+: r = -0.7; p = 0.006; Fig 3E) and positively with effector memory cells (CD4+: r = 0.6; p = 0.01; Fig 3D; CD8+; r = 0.6; p = 0.01; Fig 3F). In HIV disease, these correlations were lost in untreated subjects (Fig 3G–3J) and were not restored in the course of cART (Fig 3L–3N), except for the relationship between CD4+ naïve and Tscm cells (Fig 3K).

## Discussion

In the present study, we assessed the association between mucosal immune populations (CD4+CCR6+CD161+, CD4+CCR9+α4β7+ and Tscm subsets) and peripheral immune abnormalities persisting in the course of effective treatment for HIV infection.

We found that in HIV-infected untreated subjects with moderate immune-suppression, CD4+ cells expressing CCR6 and CD161 were depleted, possibly reflecting their permissiveness to infection [36] and redistribution from the blood to the GI tract in progressive infection [31]. CD4+CCR6+CD161+ cells in HIV-infected subjects showed increasing levels following cART introduction, yet maintained persistently lower frequencies than in controls. Consistently with this finding, we report enduring microbial translocation and gut inflammation on cART. With literature indicating the ability of CCR6+CD161+ cells to safeguard the intestinal mucosa through the production of IL-17 and IL-22 [33–35], our results suggest a partial effect of cART in correcting the homeostasis of this cell subset [31], in turn delaying the restoration of the gut barrier and promoting microbial translocation [17, 37, 38]. We also report stable levels of the Roseburia and Prevotella genera as well as the Enterobactericeae family, yet modest increases of the Lactobacillus and Bacteroides genera. These results point to the partial effect of short-term cART to correct HIV-related dysbiosis which features an outgrowth of Bacteriodes and reduction of Prevotella [39]. Our findings may be explained by the above-mentioned immune imbalances which appear to persist in the course of cART [40]; however, a longer patient follow-up is desirable in order to understand whether antiretroviral therapy may eventually modify mucosal immune homeostasis linked to microbial translocation and composition.

The frequency of CD4+CCR9+α4β7+ cells featuring a gut-homing phenotype was also significantly impaired in untreated HIV infection and correlated with the HIV RNA load and CD4+ activation. Upon cART introduction, we observed a further contraction of this subset which may be either due to their migration from the peripheral blood to the gut in the course of cART [26] or apoptosis. Although we do not have evidence to prove these hypotheses, given that CD4+CCR9+α4β7+ serve as viral targets [32], our results may point to ongoing infection and damage at mucosal sites even in the course of effective treatment, thus highlighting the role of this population in the pathogenesis of HIV disease within the gastrointestinal tract. Interestingly, recent data have demonstrated that anti-α4β7+ antibodies were able to significantly reduce lymphoid aggregates in the terminal ileum of HIV-infected subjects with mild inflammatory disease, [41] defining a possible role of anti-α4β7+ therapy for HIV eradication.

On the other hand, the absence of correlation between CD4+CCR9+α4β7+ cells and markers of T-cell activation after the introduction of treatment allow for the speculation that the repopulation of this gut-homing population at mucosal sites may be linked to decreased inflammation as well as the containment of the HIV reservoir [26]. Studies aimed at investigating the precise migration patterns of gut-homing populations and their effects on the structure of the gut barrier are warranted in humans to shed light on their contribution to HIV pathogenesis.

In line with their role in the maintenance of T cell homeostasis and cellular immunity, Tscm in HIV-uninfected controls correlated negatively with naïve subsets and positively with memory cells [42, 43]. We found that HIV-infected, untreated subjects displayed lower CD4+ and CD8+ Tscm frequencies compared to controls and no correlation was found between these pools and T-cell homeostasis. Antiretroviral treatment exerted a differential effect on CD4+ and CD8+ Tscm cells with a contraction of the former and stable frequencies of the latter. Further, a positive correlation was found between Tscm and naïve cells uniquely within the CD4+ T-cell subset. Given that Tscm populations are potentially able to migrate to the gut, our findings allow to hypothesize that CD4+ Tscm migrate to gut in the course of cART contributing to the partial restoration of peripheral T-cell homeostasis; in contrast, the restoration of the CD8+ Tscm subset, which has been linked to clinical protection in HIV infection [24, 30], may take over 12 months of cART in the clinical setting of moderate immune-depression, entailing that a lengthier follow-up of subjects enrolled in longitudinal studies is needed to explore the precise kinetics of this pool. This also holds true for the marginal impact cART appeared to have on the composition of the microbiota thus warranting new investigations on the possible relationship between cellular reservoirs and dysbiosis in HIV infection.

In conclusion, our study shows that the frequency of mucosal immune populations (CD4+CCR6+CD161+, CD4+CCR9+α4β7+ and Tscm subsets) is only partially restored in individuals starting treatment in the course of moderate immune-suppression. The persisting defects within these cell pools appear to be strictly linked to gut damage, microbial translocation and dysbiosis as well as the alteration of T-cell immunephenotypes in the peripheral blood. Longitudinal studies on paired blood and gut specimens should focus on the cART-mediated restoration and redistribution of mucosal immune populations together with their ability to serve as reservoirs of HIV.
